# Potentially inappropriate medications and medication combinations before, during and after hospitalizations: an analysis of pathways and determinants in the Swiss healthcare setting

**DOI:** 10.1186/s12913-021-06550-w

**Published:** 2021-05-28

**Authors:** Kevin Migliazza, Caroline Bähler, Daniel Liedtke, Andri Signorell, Stefan Boes, Eva Blozik

**Affiliations:** 1 Department of Health Sciences, Helsana Group, Zürich, Switzerland; 2 Department of Health Sciences and Medicine, University of Lucerne, Lucerne, Switzerland; 3 Hirslanden Group, Zürich, Switzerland; 4 Institute of Primary Care, University of Zürich, Zürich, Switzerland

**Keywords:** Drug safety, Hospitalization, Healthcare transitions, Inappropriate medication, DDI, PIM

## Abstract

**Background:**

A hospitalization phase represents a challenge to medication safety especially for multimorbid patients as acute medical needs might interact with pre-existing medications or evoke adverse drug effects. This project aimed to examine the prevalence and risk factors of potentially inappropriate medications (PIMs) and medication combinations (PIMCs) in the context of hospitalizations.

**Methods:**

Analyses are based on claims data of patients (≥65 years) with basic mandatory health insurance at the Helsana Group, and on data from the Hirslanden Swiss Hospital Group. We assessed PIMs and PIMCs of patients who were hospitalized in 2013 at three different time points (quarter prior, during, after hospitalization). PIMs were identified using the PRISCUS list, whereas PIMCs were derived from compendium.ch. Zero-inflated Poisson regression models were applied to determine risk factors of PIMs and PIMCs.

**Results:**

Throughout the observation period, more than 80% of patients had at least one PIM, ranging from 49.7% in the pre-hospitalization, 53.6% in the hospitalization to 48.2% in the post-hospitalization period. PIMCs were found in 46.6% of patients prior to hospitalization, in 21.3% during hospitalization, and in 25.0% of patients after discharge. Additional medication prescriptions compared to the preceding period and increasing age were the main risk factors, whereas managed care was associated with a decrease in PIMs and PIMCs.

**Conclusion:**

We conclude that a patient’s hospitalization offers the possibility to increase medication safety. Nevertheless, the prevalence of PIMs and PIMCs is relatively high in the study population. Therefore, our results indicate a need for interventions to increase medication safety in the Swiss healthcare setting.

## Background

In view of the growing number of elderly individuals the number of patients with one or more medically treated diseases is growing [1]. Multimorbidity represents a large challenge for drug safety as patients are in need of different medications, which increases the risk for interactions and undesired drug effects. A hospitalization phase represents an additional threat to drug safety because acute medical problems and their treatments might interact with the effects of pre-existing drugs. Consequences may be an increased morbidity or mortality of patients as well as increased healthcare costs [2–5]. However, little is known about inappropriate medication intake, especially in the context of hospitalizations in the Swiss healthcare setting. Additionally, there is a lack of knowledge about the association between inappropriate medication intake and potential determinants such as the number of additionally prescribed medications in each period, sociodemographic factors, regional aspects, or health insurance variables (e.g., whether the patient has a managed care or standard health plan).

Single drugs that might lead to adverse drug reactions that should be avoided in the elderly population are defined as potentially inappropriate medications (PIMs) [6]. Expert consensus led to PIM identification and resulted in a list of these drugs. The first list is represented by the Beers list for the USA [7], which has been updated in 2003 and 2012 [8]. Considering international differences in the availability of drugs and different prescription behaviors, several countries created their own lists such as Canada [9], France [10] or Germany (PRISCUS list) [11]. Not only single medications are part of medication safety concerns, but also certain medication combinations that might reduce expected therapy performance or even lead to adverse reactions [12–15]. A change in a drug’s effect might occur due to catabolic activation or increased concentration, possibly resulting in increased toxicity. The influence of a drug on the efficacy of another that is simultaneously taken is referred to as drug-drug interaction and is defined as potentially inappropriate medication combination (PIMC) here.

This study aims to assess the distribution of PIMs and PIMCs at three different time points (prior to, during, and after hospitalization), and the risk of receiving such inappropriate medications with every additional medication prescription when patients shift across healthcare settings. Furthermore, we explore determinants for inappropriate medication prescriptions, such as sociodemographic factors or the patient’s choice of health insurance plan.

## Methods

### Study design and study population

This retrospective analysis is based on anonymized claims data of patients with mandatory health insurance at the Helsana Group, a leading health insurer in Switzerland, currently covering about 1.2 million individuals, as well as on data from the Hirslanden Swiss Hospital Group, comprising 16 private hospitals distributed across the country but with a concentration of hospital beds in the canton of Zürich. These hospitals were included in the hospital list of the canton where the hospital is situated, so that all insured persons with basic health insurance had primary access to these hospitals. All adult persons who were hospitalized in a Swiss private hospital group in the year 2013 and who were enrolled with Helsana basic health insurance in the 6 months preceding and following the hospitalization were included in the present study. This data linkage has been approved by the cantonal ethics committee of the Canton of Zurich (KEK-ZH-Nr. 2014–0261). In compliance with the Swiss Federal Law on data protection, all data were anonymized. Because the data were retrospective, pre-existing, and de-identified, no informed consent was needed.

The study population consists of 5′637 patients aged 65 years and older who were hospitalized in an acute hospital of the Hirslanden Swiss Hospital Group in the year 2013 and who had mandatory health insurance during 2013 at the Helsana Group. Patients younger than 65 years were exempted from the analysis since a negligible proportion (1.2%) of the study population received at least one PIMC in this age group and PIMs are by definition identified for a target population aged 65 years and older. Re-hospitalizations in the same year were not considered, therefore an individual was only included once.

### Measures

We assessed medication safety by means of PIMs and PIMCs at three different time points: in the quarter year prior to hospitalization (pre-hospitalization, Q2), during hospitalization (peri-hospitalization, H), and during the quarter following the hospitalization period (post-hospitalization, Q3). As hospitalizations in 2013 were considered, pre- and post-hospitalization periods could also include data of 2012 and 2014. In order to reconstruct the medication sequence during the continuum of care, information from the hospital on medication administration was merged with our data on prescriptions outside the hospital setting (Q2,Q3). Both our data and the hospital group use the standardized Anatomical Therapeutic Chemical (ATC) Classification System Level 5 (e.g., ciprofloxacin (J01MA02)) for naming drug chemical entities. This ensures consistent drug nomenclature across the continuum of care (Q2,H,Q3). Given the high degree of comparability between the German and the Swiss setting in terms of drug availability, PIMs were identified using a list of ATC codes based on the German PRISCUS list for the present study. Thereby, each active substance from this list was attributed to one or multiple level 5 ATC codes using the WHO ATC/DDD Index. Compared to PIMs, there are significant differences and controversies about what should be considered a core list with respect to PIMCs [16–21]. For the present work, PIMCs were identified using a list of potentially inappropriate drug pairs derived from the Swiss Drug Compendium, as this database is adapted to Swiss drug prescribing practices and has been used in prior studies (www.compendium.ch) [22, 23]. EPha.ch, a Spin-off from the University of Zurich, addressed this issue and created a software for physicians and pharmacists which identifies patients with inappropriate medication combinations based on the Swiss drug compendium [24]. PIMCs were defined as the prescription of a drug that might interact with another medication a patient has been taking in the preceding 3 months. For instance, patients with a new prescription of antithrombotic agents and who were prescribed acetylsalicylic acid within the preceding 3 months were classified as PIMC users. In order to assess the most frequently prescribed PIMCs at the level of main therapeutic groups, these ATC codes were transformed into ATC level 2 (e.g. bemiparin (B01AB12) was classified as antithrombotic agent (B01)).

As our analysis estimated the risk of PIM and PIMC prescriptions by each additional prescription when patients shift across healthcare settings, we assessed medication increments by comparing total medications in each period to those of the preceding period. Sociodemographic variables included sex and age group (65–69, 70–74, 75–79, 80+ years), and language region was included as regional variable. Since the Hirslanden Hospital Group is predominantly represented in Zurich and given the scarcity of patients residing in other cantons, the analysis did not account for regional variation on the cantonal level, but rather differentiates between French and German speaking regions.

In Switzerland, basic health care is covered by mandatory health insurance with a standardized coverage package for in- and outpatient care as well as listed medical products. The health insurance market is regulated as managed competition. Patients pay health insurance premiums (community-rated) and there is additional cost-sharing composed of deductibles and copayments. The latter is a charge of 10% of the costs per patient in 1 year and cannot exceed 700 Swiss francs/year. The basic level of deductibles is CHF 300 per year, but individuals can opt for a higher deductible plan to receive a lower premium. In addition to mandatory health insurance, patients might opt for supplementary hospital insurance. This covers further comforts such as semiprivate or private wards, free doctor selection, and facilitated access for elective procedures. Regarding the patients’ type of health insurance plan, being enrolled in a managed care model, having supplementary hospital insurance, and having a low deductible (CHF 300, 500) were used as binomial variables in the analysis.

Residence in the French-speaking region of Switzerland, sociodemographic variables and insurance variables were obtained from the insurance claims data. Finally, the analysis also included 17 different inpatient diagnosis groups, derived from the ICD-10 classification system, which have been obtained from the hospital records as these diagnosis codes have been assigned to patients during their hospital stay: Neoplasia; diseases of the circulatory system; diseases of the respiratory system; diseases of the digestive system; diseases of the musculoskeletal system and connective tissue; diseases of the urogenital system; and injuries, poisoning and certain consequences of external causes. Due to comparably low occurrences, the following diseases were grouped as ‘others’: Infections and parasitosis; diseases of the blood and blood-forming organs and immune system; endocrine, nutritional and metabolic diseases; mental and behavioral disorders; diseases of the nervous system; diseases of the eye and eye appendages; diseases of the ear and mastoid process; diseases of the skin and subcutaneous tissue; diseases not elsewhere classified; and factors influencing health status.

### Statistical analysis

**Fig. 1:**
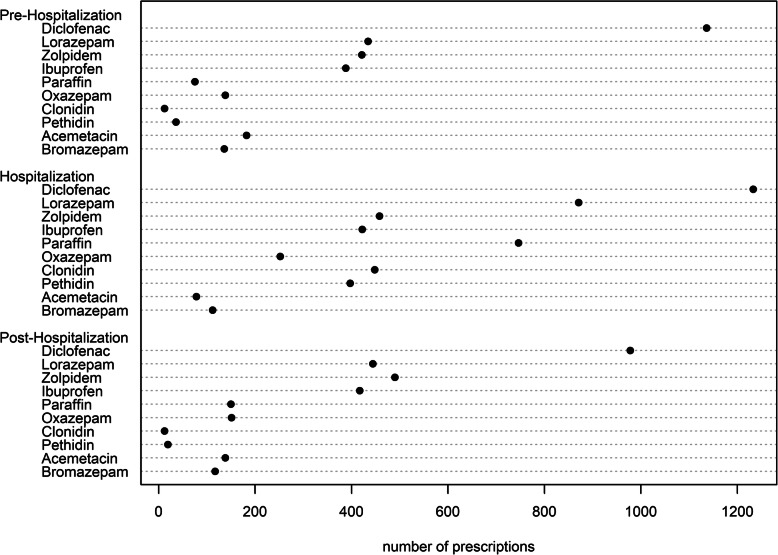
Dotplot shows the most frequently prescribed PIMs, vertically ordered by decreasing number of prescriptions over the observation period

**Fig. 2:**
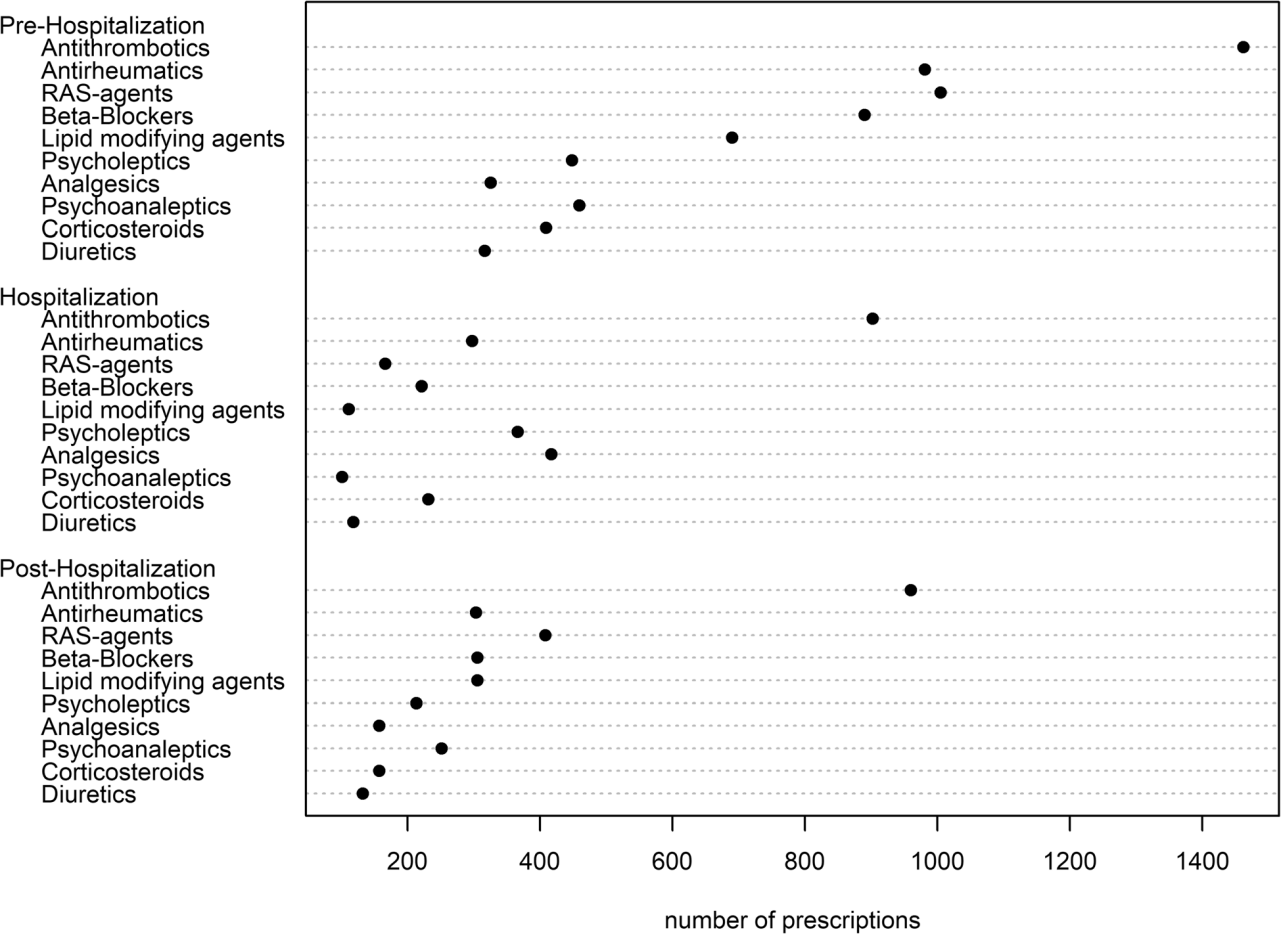
Dotplot shows the most frequently prescribed PIMCs, vertically ordered by decreasing number of prescriptions over the observation period

**Fig. 3:**
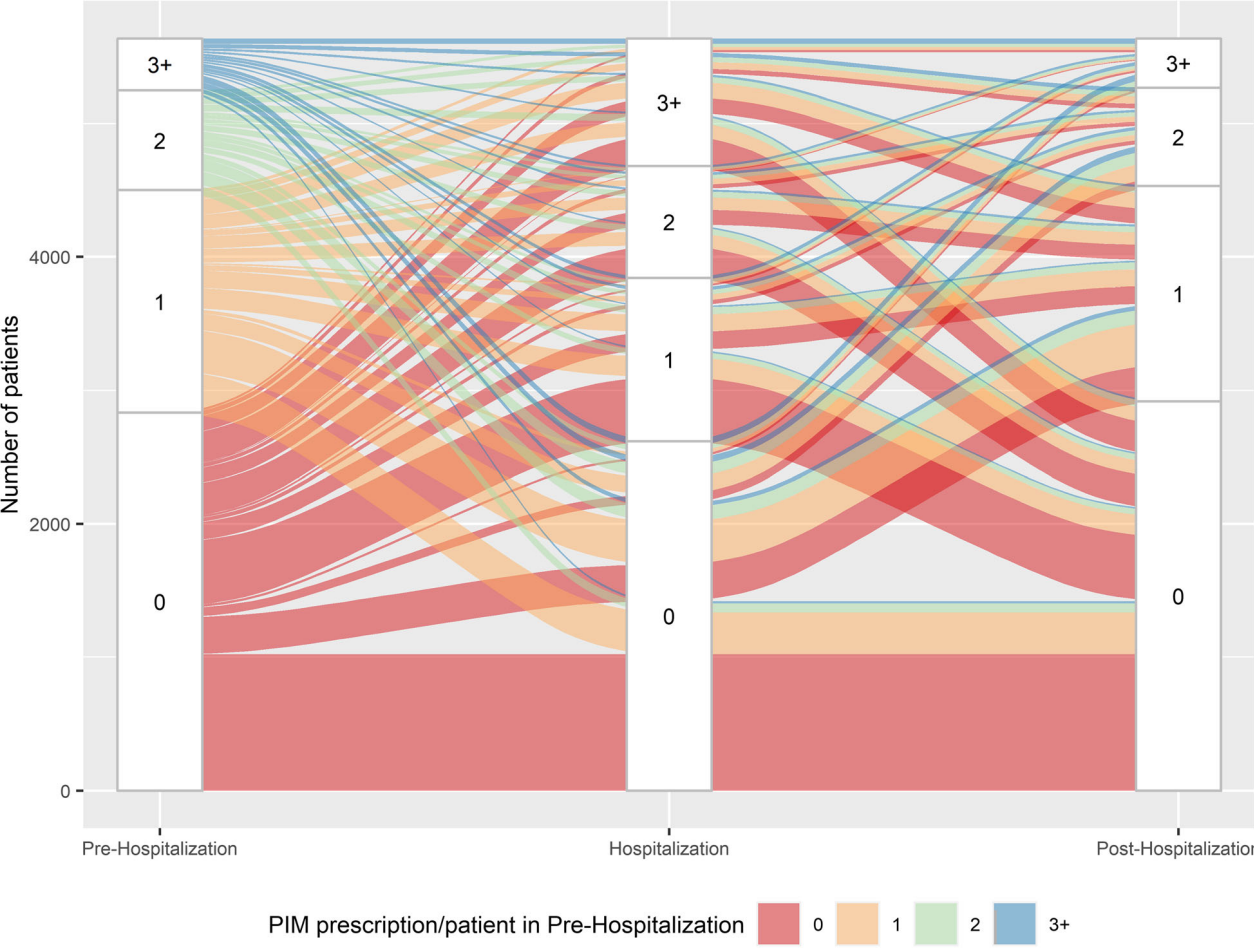
PIM use in the pre-, peri- and post-hospitalization period

**Fig. 4:**
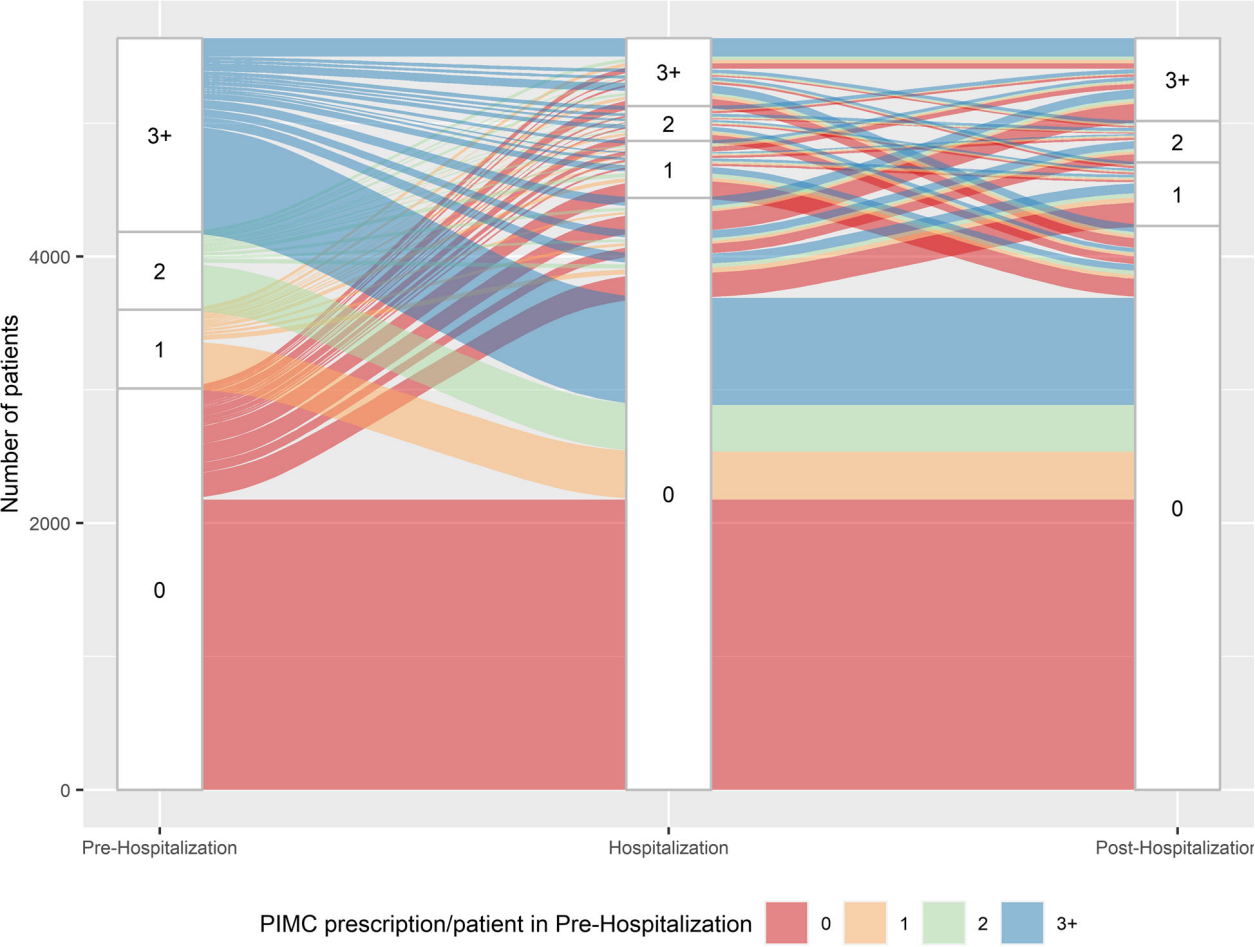
PIMC use in the pre-, peri- and post-hospitalization period

Descriptive statistics were used to analyze the differences of PIM and PIMC use across patient characteristics and across different time points (Tables 1 and 2). Dotplots show the most frequently prescribed PIMs and PIMCs (Figs. 1 and 2). Alluvial plots depict changes in the number of PIMs and PIMCs across the continuum of care (Figs. 3 and 4) [25]. Cochran-Tests were used as first step to test for overall differences in the number of PIM and PIMC users over the entire observation period (Q2, H, Q3). If the hypothesis of equal distribution was rejected, post-hoc McNemarTests were used to assess differences between all observation periods (Q2 and H, H and Q3, Q2 and Q3). Furthermore, Friedman-Tests were used as a first approach to test for global differences in total PIMs and PIMCs count and tested the same group at several points in time (Q2, H, Q3) without further stratification. Again, if significant differences were observed, Pairwise Wilcoxon Rank Sum Tests were used to evaluate differences in the numbers of PIM and PIMC prescriptions across all observation periods (Q2 and H, H and Q3, Q2 and Q3). In addition, the most frequently added and most frequently reduced PIM prescriptions during each observation period (Q2,H,Q3) were described in Table 3.

**Table 1:** Characteristics of the study population

Variables	Total	PIM use	PIMC use
Observations, n (%)	5′637	4′616 (81.9)	3′461 (61.3)
Patients with additional medication prescriptions, n (%)			
Pre-Hospitalization	3′132 (55.6)	2′610 (56.5)	958 (27.7)
Hospitalization	3′331 (59.1)	2′924 (63.3)	1′563 (45.2)
Post-Hospitalization	2′467 (43.8)	2′040 (44.2)	958 (27,7)
*Sociodemographics*, n (%)			
Age group by male sex			
65–69 years	682 (26.0)	516 (25.5)	349 (18.7)
70–74 years	665 (25.4)	511 (25.2)	424 (22.7)
75–79 years	530 (20.2)	408 (20.1)	412 (22.1)
80+ years	743 (28.4)	592 (29.2)	680 (36.5)
Age group by female sex			
65–69 years	709 (23.5)	613 (23.7)	357 (22.4)
70–74 years	732 (24.3)	631 (24.4)	400 (25.1)
75–79 years	620 (20.6)	523 (20.2)	345 (21.6)
80+ years	956 (31.7)	822 (31.7)	494 (31.0)
Female sex	3′017 (53.5)	2′388 (56.3)	1′865 (53.9)
French speaking region	559 (9.9)	461 (10.0)	312 (9.0)
*Insurance variables*, n (%)			
Low deductible (300,500)	5′015 (89.0)	4′150 (89.9)	3′191 (92.2)
Managed care	2′065 (36.6)	1′696 (36.7)	1′212 (35.0)
Supplementary hospital insurance	3′267 (58.0)	2′636 (57.1)	1′994 (57.6)
Diagnosis group, n (%)			
Neoplasia	484 (8.6)	422 (9.1)	255 (7.4)
Circulatory system	904 (16.0)	661 (14.3)	693 (20.0)
Respiratory system	118 (2.1)	93 (2.0)	80 (2.3)
Digestive system	409 (7.3)	348 (7.5)	223 (6.4)
Musculoskeletal system and connective tissue	1′068 (18.9)	986 (21.4)	674 (19.5)
Urogenital system	267 (4.7)	212 (4.6)	131 (3.8)
Injuries, poisoning and certain other consequences	274 (4.9)	234 (5.1)	170 (4.9)
Other diagnosis	427 (7.6)	325 (7.0)	256 (7.4)

**Table 2:** PIM and PIMC use in the pre-, peri- and post-hospitalization period

	Pre-hospitalization	Hospitalization	Post-hospitalization
**PIMs**			
User, n (%)	2′804 (49.7)	3′019 (53.6)	2′719 (48.2)
PIMs/Patient, mean (SD)	0.80 (1.02)	1.18 (1.53)	0.78 (1.04)
Median (25,75%)	1 (0,1)	1 (0,2)	1 (0,1)
Prescriptions, n (%)	4′499 (9.9)	6′673 (9.2)	4′399 (9.9)
**PIMCs**			
User, n (%)	2′629 (46.6)	1′199 (21.3)	1′409 (25.0)
PIMCs/Patient, mean (SD)	1.52 (2.18)	0.67 (1.83)	0.69 (1.56)
Median (25,75%)	1 (0,3)	0 (0,0)	0 (0,0)
Prescriptions, n (%)	8′575 (18.9)	3′756 (5.2)	3′894 (8.7)

**Table 3:** most added and most reduced PIM prescriptions in the pre-, peri- and post-hospitalization period

**From Pre-hospitalization (Q2) to Hospitalization (H)**							
**Most added**	**Total prescriptions**		**Changes (n)**	**Most reduced**	**Total prescriptions**		**Changes (n)**
**ATC**	**Q2**	**H**	**∆Q2/H**	**ATC**	**Q2**	**H**	**∆Q2/H**
Paraffin	75	746	+ 671	Ketoprofen	125	1	-124
Lorazepam	434	871	+ 437	Acemetacin	182	78	− 104
Clonidine	12	448	+ 436	Etoricoxib	98	8	−90
**From Hospitalization (H) to Post-hospitalization (Q3)**							
**Most added**	**Total prescriptions**		**Changes (n)**	**Most reduced**	**Total prescriptions**		**Changes (n)**
**ATC**	**H**	**Q3**	**∆H/Q3**	**ATC**	**Q2**	**H**	**∆H/Q3**
Ketoprofen	1	97	+ 96	Paraffin	746	150	− 596
Sodium picosulfate	73	138	+ 65	Clonidine	448	12	−436
Acemetacin	78	138	+ 60	Lorazepam	871	444	− 427
**From Pre-hospitalization (Q2) to Post-hospitalization (Q3)**							
**Most added**	**Total prescriptions**		**Changes (n)**	**Most reduced**	**Total prescriptions**		**Changes (n)**
**ATC**	**Q2**	**Q3**	**∆Q3/Q2**	**ATC**	**Q2**	**Q3**	**∆Q3/Q2**
Paraffin	75	150	+ 75	Diclofenac	1136	978	− 158
Zolpidem	421	490	+ 69	Etoricoxib	98	50	−48
Sodium picosulfate	87	138	+ 51	Acemetacin	182	138	−44

Given the skewness of the distribution and the excessive number of zeros, a zero-inflated Poisson (ZIP) regression model was used to predict the number of PIMs and PIMCs:

1 $$ P\left(Y=y\right)=\left\{\begin{array}{cc}\ \left(1-\pi \right)+\pi \bullet g(0)& if\ y=0\\ {}\pi \bullet g(y)& if\ y>0\end{array}\right. $$

where *π* ∈ [0, 1] denotes the inflation parameter, and *g*(*y*) denotes the probability function of the Poisson distribution. The zero-inflation parameter was specified as

2 $$ {\displaystyle \begin{array}{c}\log \left(\frac{\pi }{1-\pi}\right)={\beta}_0+{\beta}_1\bullet ADDQ2+{\beta}_2\bullet ADDH+{\beta}_3\bullet ADDQ3+{\beta}_4\bullet AGE\\ {}+{\beta}_5\bullet FEMALE+{\beta}_6\bullet AGE\bullet FEMALE+{\beta}_7\bullet LATINCT+{\beta}_8\bullet LOWD\\ {}+{\beta}_9\bullet MC+{\beta}_{10}\bullet SUPPLH+{\beta}_{11-18}\bullet {DIAG}_{1-8}\end{array}} $$

and the mean function of the Poisson distribution was specified as

3 $$ {\displaystyle \begin{array}{c}\log {E}_g(Y)={\beta}_0+{\beta}_1\bullet ADDQ2+{\beta}_2\bullet ADDH+{\beta}_3\bullet ADDQ3+{\beta}_4\bullet AGE\\ {}+{\beta}_5\bullet FEMALE+{\beta}_6\bullet AGE\bullet FEMALE+{\beta}_7\bullet LATINCT+{\beta}_8\bullet LOWD\\ {}+{\beta}_9\bullet MC+{\beta}_{10}\bullet SUPPLH+{\beta}_{11-18}\bullet {DIAG}_{1-8}\end{array}} $$

The used dependent and independent variables were:

- Y Number of PIMs or PIMCs
- ADDQ2 Number of additional medications in Q2 compared to previous time period (Q1)
- ADDH Number of additional medications in H compared to previous time period (Q2)
- ADDQ3 Number of additional medications in Q3 compared to previous time period (H)
- AGE Age of patient, in age groups 65–69, 70–74, 75–79, 80+
- FEMALE dummy variable, 1 if female, 0 if male
- LATINCT dummy variable, 1 if residence in Latin canton, 0 if not
- LOWD dummy variable, 1 if patient has low deductible (300,500), 0 if not
- MC dummy variable, 1 if patient is enrolled in managed care plan, 0 if not
- SUPPLH dummy variable, 1 if patient has supplementary hospital insurance, 0 if not
- DIAG 8 different binomial variables for the inpatient diagnosis (therefore, multiple diagnoses are possible for each individual), 1 if patient has the diagnosis, 0 if not

**Fig. 5:**
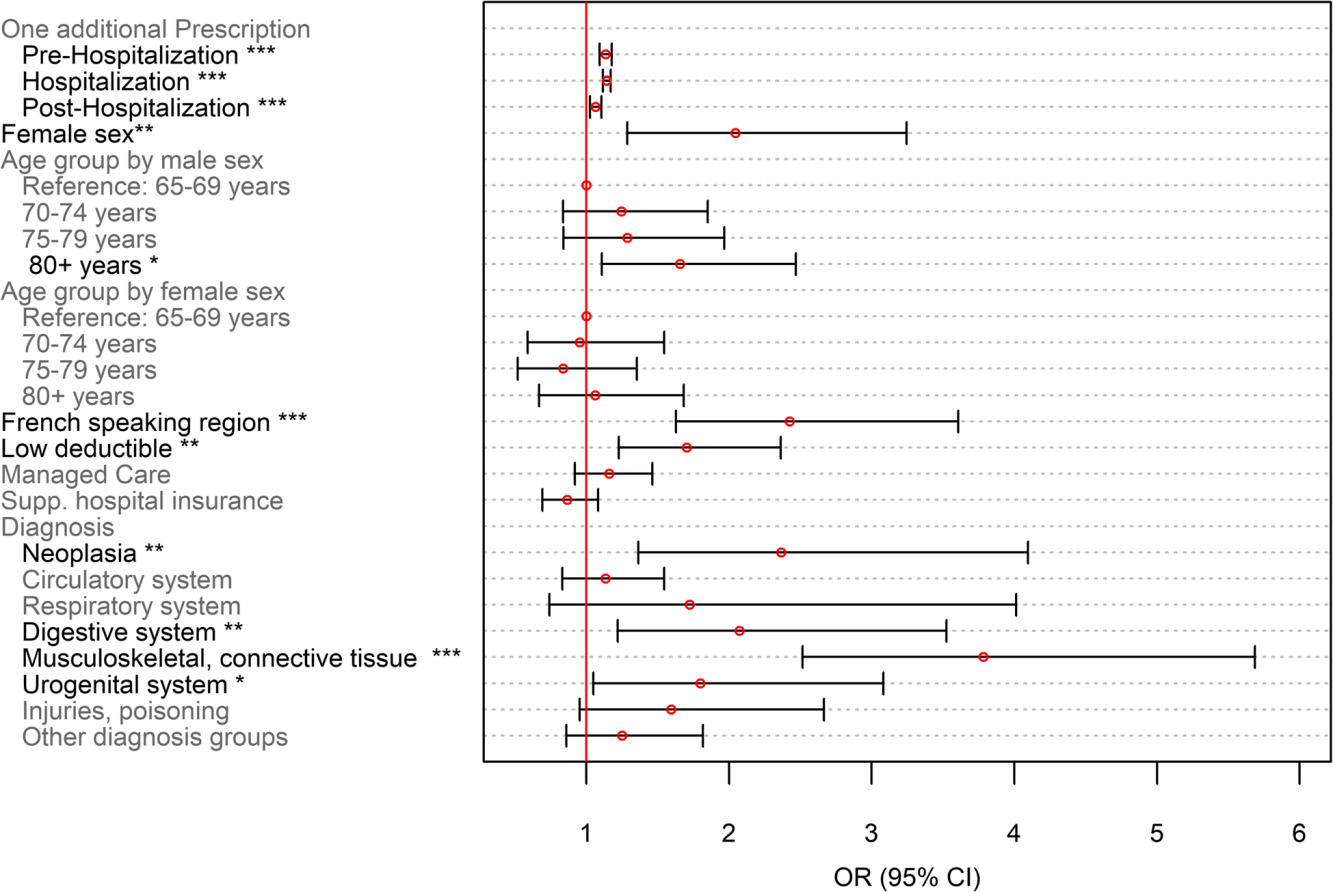
Binomial model on PIMs as part of the zero-inflated poisson regression model on the number of PIMs. *N* = 5′637; *: *p* < 0.05; ** *p* < 0.01; ***: *p* < 0.001

**Fig. 6:**
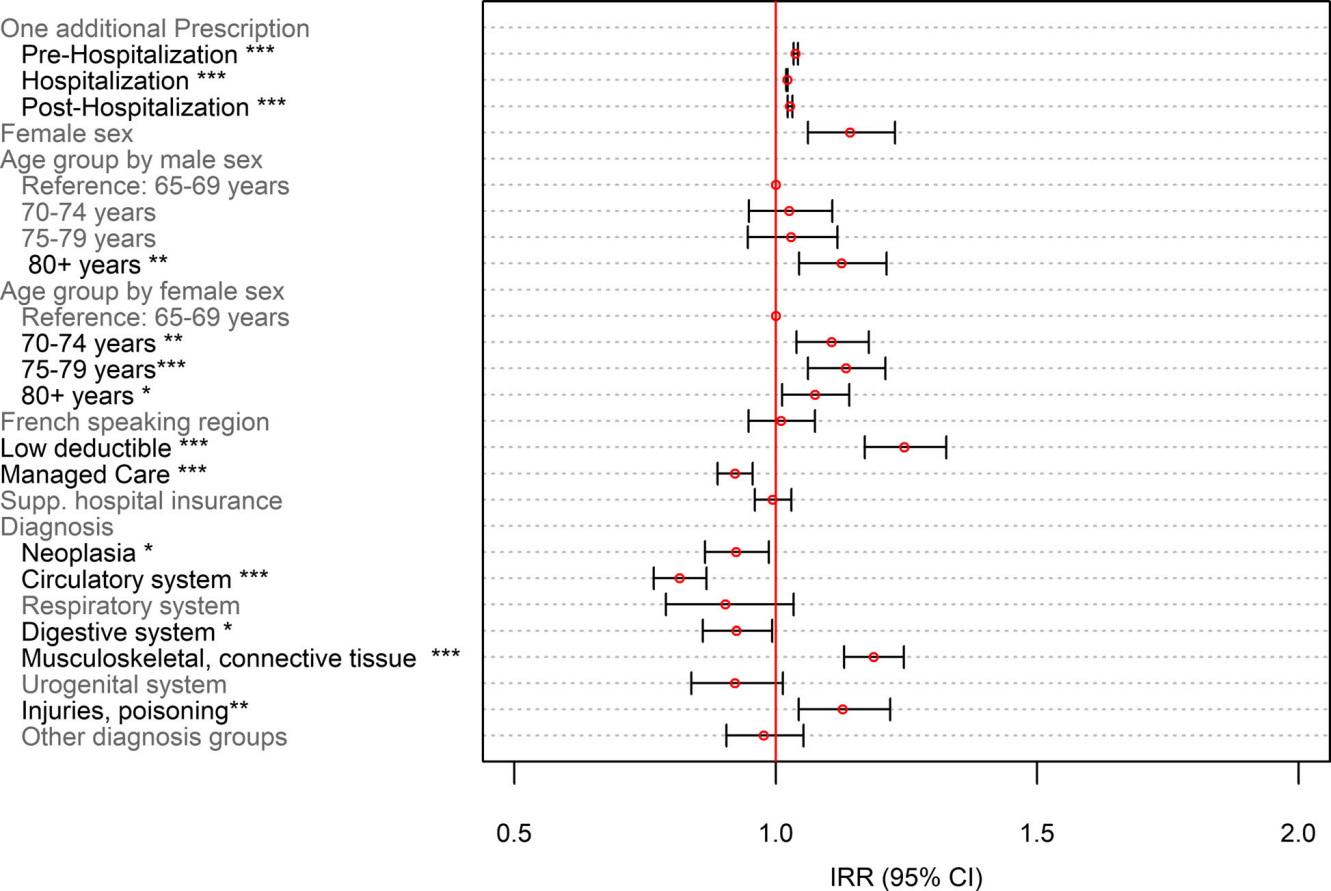
Poisson model on the number of PIMs as part of the zero-inflated poisson regression model on the number of PIMs. *N* = 5′637; *: *p* < 0.05; ** *p* < 0.01; ***: *p* < 0.001

**Fig. 7:**
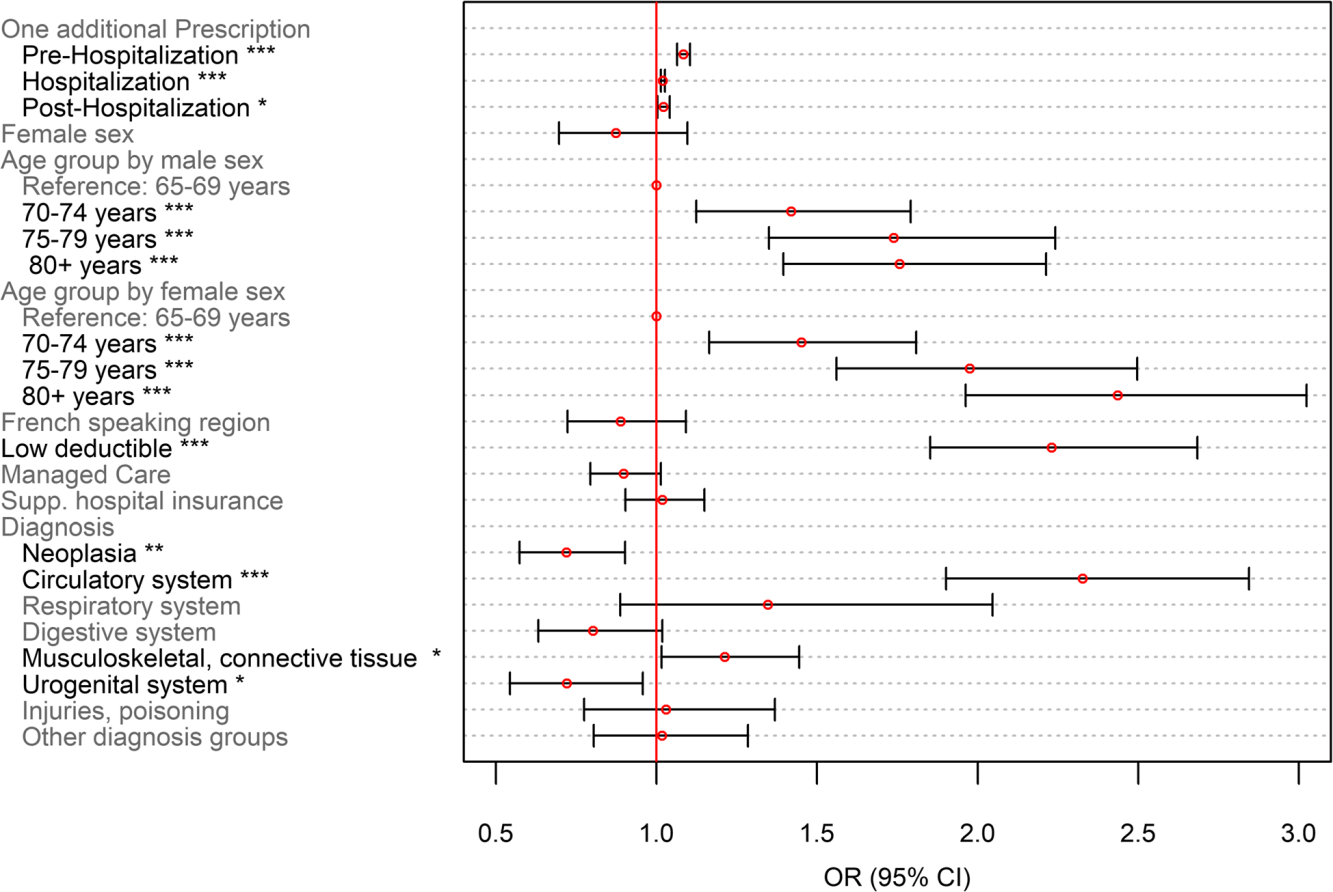
Binomial model on PIMCs as part of the zero-inflated poisson regression model on the number of PIMCs. *N* = 5′637; *: *p* < 0.05; ** *p* < 0.01; ***: *p* < 0.001

**Fig. 8:**
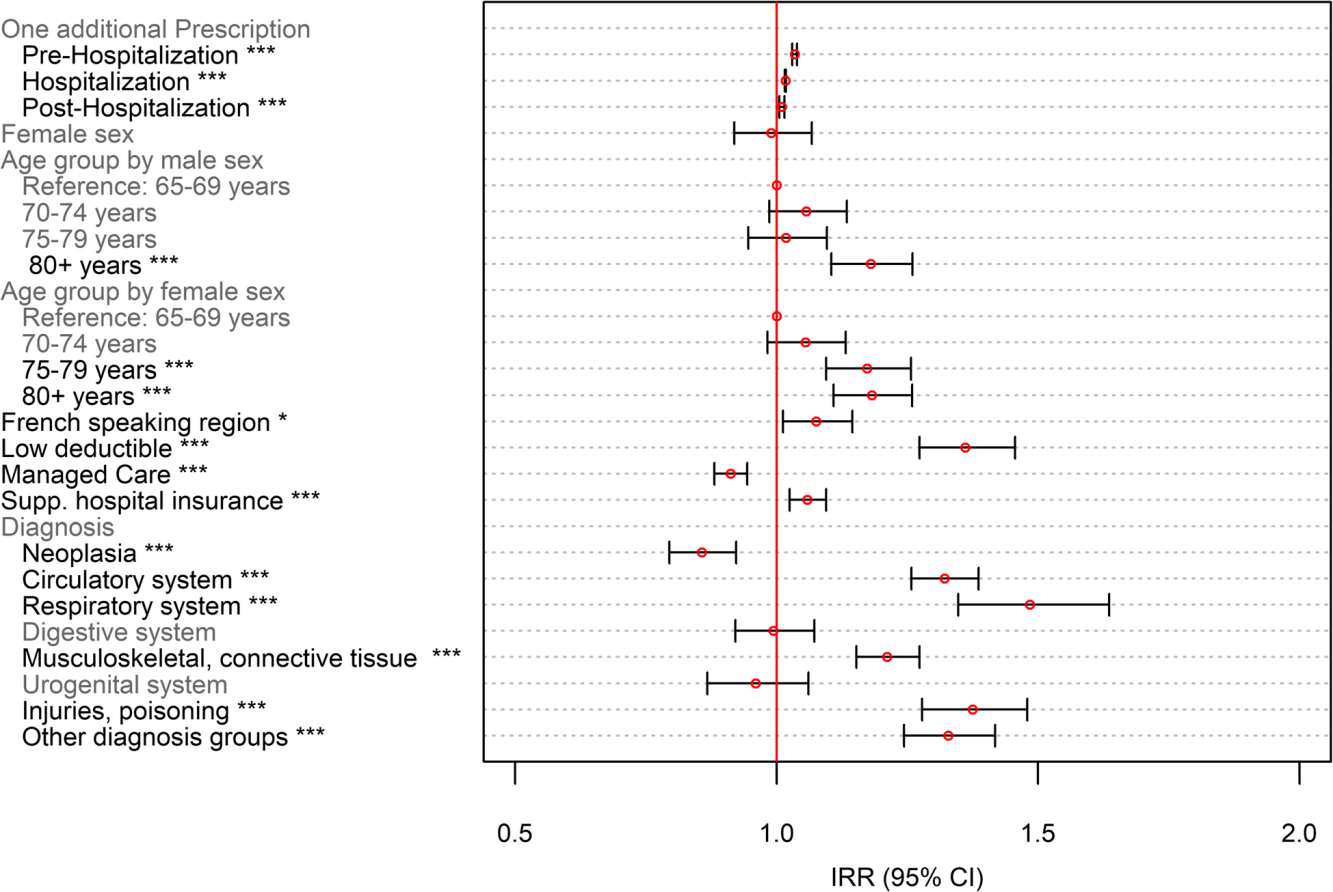
Poisson Regression on the number of PIMCs as part of the zero-inflated poisson regression model on the number of PIMCs. *N* = 5′637; *: *p* < 0.05; ** *p* < 0.01; ***: *p* < 0.001

Odds ratios (OR) were shown for dichotomous outcomes (Figs. 5 and 7), whereas incidence risk ratios (IRRs) are depicted for the number of PIMs and PIMCs (Figs. 6 and 8). ORs were calculated for the ratio of the probability that a patient received at least one PIM or PIMC versus the probability that this event will not occur. These were obtained by inverting the coefficients (*e*^−*β*^) of the classical binomial part of the Poisson regression model. For the interpretation of the coefficients of both models, the following remarks should be considered: The term “occurrence” refers to the binomial model. For instance, “PIM occurrence” addresses the scenario that a patient received at least one PIM. IRRs relate to non-zero-outcomes. For the description of those coefficients, we often used the notion of “number of prescriptions”.

Our model included sex related differences in age-dependency with regards to PIMs and PIMCs using interaction terms. In our models, reference groups were chosen and indicated in the result section (Figs. 5, 6, 7 and 8). Regarding the medical condition, patients were assigned the value 1 in those diagnostic variables for which they demonstrated the corresponding diagnosis. Patients without the diagnosis were chosen to constitute the reference group (value 0) and represented the larger category in all diagnoses. This facilitated the assessment of PIM and PIMC prescriptions in patients who had a specific diagnosis compared to not having the medical condition. The analyses were conducted using R statistics, version 3.5.0.

## Results

Of the 5′637 people representing the study population, 53.5% were female. The mean (+SD) age was 75.2 years (±7.1) in men and 75.9 years (±7.3) in women. Overall, the number of patients with at least one additional medication prescription compared to the preceding period amounted to 5′248 (93.1%) and was composed of 3′132 (55.6%) patients who had experienced an increase from the quarter year prior to pre-hospitalization to pre-hospitalization, 3′331 (59.1%) patients had increases in prescriptions after hospital admission, and 2′467 (43.8%) after discharge. Most frequently, patients had a diagnosis of the musculoskeletal system and the connective tissue, or of the circulatory system. Throughout the entire observation period, 81.9% of all patients received at least one PIM and 61.3% received at least one PIMC. Characteristics of the study population by PIM and PIMC use are shown in Table 1.

The active substance diclofenac was the most frequently prescribed PIM (Fig. 1), and antithrombotic agents were the most frequently prescribed drugs possibly leading to an interaction with pre-existing medications (Fig. 2). The PIM and PIMC use in the pre-, peri- and post-hospitalization period are presented in Table 2.

The number of PIM users increased from the pre-hospitalization (*n* = 2′804) to the hospitalization period (*n* = 3′019, *p* < 0.001) and decreased again after discharge (*n* = 2′719) compared to the hospitalization period (*p* < 0.001). When assessing the change of the PIM users from the pre- to the post-hospitalization period, only a slight decrease could be observed (*p* = 0.04). In contrast, the number of patients being prescribed at least one PIMC decreased by nearly 56% from the pre-hospitalization (*n* = 2′629) to the hospitalization period (*n* = 1′199, *p* < 0.001), whereas it marginally increased after discharge as compared to the hospitalization period (*n* = 1′409, *p* < 0.001). The average number of PIMs per patient changed over the continuum of care with highest values during hospitalization (1.18). Compared to both, the pre- (0.80) and the post-hospitalization (0.78) phase, patients had a significantly higher average number of PIMs during hospital care. However, no significant changes were observed comparing the periods before hospital admission with the period after hospital discharge (*p* = 0.11). On the other hand, the average number of PIMCs per patient decreased significantly from the pre-hospitalization (1.52) to the hospitalization period (0.67, *p* < 0.001) and increased only marginally after discharge as compared to the hospitalization period (0.69, *p* < 0.001). However, comparing the mean number of PIMCs per patient between the pre-hospitalization and the post-hospitalization period, this resulted in a significant decrease.

Figure 3 shows changes of PIMs per patient across the continuum of care. A higher proportion experienced an increase in single inappropriate medications after hospital admission: During hospitalization, 2′197 (39.0%) patients had a higher PIM count compared to the pre-hospitalization period (red strips that move into category 1,2,3+ in H/orange strips that move into category 2,3+ in H/green strips that move into category 3+ in H), whereas a lower number of PIMs was observed in 1′548 (27.5%) patients (blue strips that move into category 0,1,2 in H/green strips that move into category 0,1 in H/orange strips that move into category 0 in H). After discharge, 1′482 (26.3%) patients had an increase, and 2′253 (40.0%) patients experienced a decrease in the number of PIMs. Likewise, changes in PIMCs are illustrated in Fig. 4. With regards to PIMCs, a reduction in potential medication errors was observed for a higher proportion of patients: During the transition from the pre-hospitalization to the hospitalization period, 724 patients (12.9%) experienced an increase (red strips that move into category 1,2,3+ in H/orange strips that move into category 2,3+ in H/green strips that move into category 3+ in H) and 2′211 (39.2%) a decrease in PIMCs (blue strips that move into category 0,1,2 in H/green strips that move into category 0,1 in H/orange strips that move into category 0 in H). After hospital discharge, 1′009 patients (17.9%) had a higher number and 799 (14.2%) a lower number of PIMCs compared to the preceding time period. Comparing Figs. 3 and 4, further illustrates the increased occurrence of PIM users compared to PIMC users during and after hospitalization.

Looking at PIM prescriptions in more detail, the strongest changes were found for paraffin, lorazepam and clonidine as they increased significantly during the transition from pre-hospitalization period to hospitalization, whereas the discontinuation of those active substances was similarly accentuated after hospital discharge (Table 3). This indicates that these medications are mainly prescribed during hospitalization. By contrast, the number of prescriptions for ketoprofen, acemetacin, and etoricoxib were reduced during the hospital stay. The most pronounced increase in PIM prescriptions from pre- to post-hospitalization was observed for paraffin (again), zolpidem, and sodium picosulfate. In contrast, patients were prescribed less diclofenac, etoricoxib, and acemetacin after hospitalization, with the reduction most pronounced for diclofenac.

As far as the prescription of at least one PIM throughout the entire observation period is concerned, the main explanatory variables were additional medication prescriptions during healthcare transitions, female sex, residence in a French speaking region and having a low deductible (Fig. 5). Every additional medication prescription increased the odds of receiving at least one PIM independently of the period, but an increment in medications in the hospital setting showed the strongest association (OR = 1.143 [1.116–1.170], *p* < 0.001). Furthermore, the following four diagnosis groups were associated with increased PIM occurrence: neoplasia, as well as diseases of the digestive system, the musculoskeletal system and connective tissue, and the urogenital system.

An increase in the number of medications was associated with an increased number of PIMs ranging from 2.2% after hospital admission to 3.8% in the pre-hospitalization period (Fig. 6). Overall, the female population had a 14.2% higher number of PIMs compared with men. The number of PIMs increased with increasing age in women. On the contrary, only men aged 80 years and older had a 12.6% higher number of PIMs compared with men in the youngest age group. Being in a managed care model was associated with lower PIM prescriptions (IRR = 0.922 [0.889–0.956], *p* < 0.001), whereas having a low deductible was associated with a higher number of PIMs (IRR = 1.246 [1.170–1.326], *p* < 0.001). Neoplasia, diseases of the circulatory, and of the digestive system were associated with decreased number of PIMs, whereas diseases of the musculoskeletal system and connective tissue, and injuries, poisoning and certain other consequences of external causes were associated with higher PIM counts.

Regarding PIMC use, receiving additional medications was associated with increased odds of PIMC occurrence, with the highest increment of 8.4% in the pre-hospitalization period (Fig. 7). Being in a higher age group in men and women, and having a low deductible increased the odds of receiving at least one PIMC, whereas no significant differences in PIMC occurrence were observed between men and women. Patients with neoplasia and diseases of the urogenital system were associated with a decreased, while patients with diseases concerning the circulatory system or the musculoskeletal and connective tissue were associated with increased odds of PIMC prescriptions.

Each additional medication following a healthcare transition was associated with an increased number of PIMCs (Fig. 8). Thereby, the effect of each additional medication weakened across the continuum of care as the incident risk ratio decreased from 3.5% in the pre-hospitaliation period to 1.0% after hospital discharge. No significant gender-related difference in PIMC prescriptions was evident. Higher age increased the number of PIMC prescriptions. The number increased by 17.5% in women aged 75–79 years and by 18.4% in women aged 80 years and older, whereas men aged 80 years and older had 18.0% higher PIMC counts, compared with the youngest age group. Being in a managed care model was associated with a decrease in the number of PIMCs by 8.8%, while having a low deductible, supplementary hospital insurance, and living in a French speaking region increased PIMC prescriptions. Diseases of the digestive and the urogential system had no influence on the number of PIMCs, while neoplasia were negatively and all other diagnoses were positively associated with the number of PIMC use.

In sum, factors, that were consistently and positively associated with increased PIM and PIMC prescriptions were the increase in total medication prescriptions compared to the preceding period, increased age, and having a low deductible. Women had higher chances of receiving PIMs, whereas the likelihood of receiving PIMCs was not affected by gender. Managed care was strongly associated with a decrease in the number of PIMs and PIMCs. French speaking regions were significantly associated with increases in PIMs and PIMCs in the count models, whereas having supplementary insurance showed only an association with increased number of PIMCs, but not with PIMs.

## Discussion

In the present analysis, we examined the distribution of PIMs and PIMCs at three different time points (prior to, during, and after hospitalization). Firstly, we found a high overall prevalence of PIM (81.9%) and PIMC (61.3%) users in this elderly population cohort. Secondly, the present study shows that the prevalence of PIMC users decreased across the three different time periods (from 46.6% in the pre-hospitalization period, to 21.3% during hospitalization and to 25% in the quarter after discharge), whereas PIM users decreased only marginally between the pre- (49.7%) and post-hospitalization period (48.2%), with highest proportions found while patients were hospitalized. Thirdly, additional medication prescriptions and increasing age were associated with increased inappropriate medication and medication combinations use, whereas managed care was mainly associated with fewer inappropriate medications or medication combinations.

Comparing our results to those of previous studies, we found an increased prevalence of PIMs and PIMCs. Blozik et al. estimated the quarter year prevalence of PIMs in the population aged older than 65 years to be 21.1% [6]. Another Swiss study estimated PIM prevalence among patients in nursing homes to be 59.5% if the PRISCUS list is considered [26]. PIM prevalence in home care settings were found to be 19.8% in the European population aged 65+, whereby substantial differences were observed between Western Europe (mean 15.8%, ranging from 5.8% in Denmark to 26.5% in Italy) and Eastern Europe (41.1% in Czech Republic) [27]. A German study found that 25% of all patients aged 65+ had at least one annual PIM prescription [28]. Egger et al. explored PIM prescriptions in a 700-bed teaching hospital in Switzerland during 2004 by means of Beers criteria and found that 16% of patients in general medical wards and 20.8% of geriatric patients received at least one PIM [29]. These discrepancies in PIM prevalence might be due to different observation periods and the use of different underlying PIM screening methodology. Moreover, the higher PIM prevalence shown in our analysis might result due to the fact that we included hospitalized patients who presumably had higher medical needs compared to those of the total Swiss population [6].

Furthermore, we found an increased number of PIMC users compared to a previous Swiss study that explored potential drug-drug interactions (DDI) in the outpatient setting and identified potential DDIs in 1.2% of men and 1.3% of women [30]. The substantially lower prevalence compared to our study can be explained by the different underlying databases used to identify PIMCs and the low sensitivity of the Pharmavista software used in their study. Indeed, a previous Swiss study showed that Pharmavista only identifies 9.1% of all clinically relevant interactions [31]. Furthermore, our analysis is based on an elderly population, whereas the previous study estimated drug-drug interaction among all age groups, including less morbid younger age groups, therefore, resulting in lower proportions of individuals with at least one PIMC prescription. Moreover, differences in medication regimen in the outpatient compared to the inpatient setting might further explain discrepancies in PIMCs. According to a meta-analysis and systematic review, 33% of all hospitalized general patients received at least one PIMC, [32] whereby PIMC prevalence ranged from 16.3% [33] to 71.1% [34].

Regarding PIM and PIMC prescriptions across the three different time periods, we found that an increase in medication prescriptions increased the probabilities of receiving PIMs and PIMCs. These basic findings are consistent with research showing that polypharmacy is highly associated with PIMs and PIMCs [35–37]. In our analysis, chances of receiving PIMCs were found to be higher prior to hospital admission compared to after discharge. These findings suggest that medications are carefully re-examined in the context of a hospitalization. This result of lower PIM prevalence after hospital discharge is consistent with previous research, in which the PIM prevalence decreased from 52% in the pre-hospitalization to 42% at discharge [38]. Though, some PIM prescriptions reached peak prescription levels during hospitalization (paraffin, lorazepam and clonidine), which could be explained by the fact that patients were in an acute medical situation. For example, clonidine is often used in the acute treatment of arterial hypertension. Many anti-inflammatory and anti-rheumatic PIMs (ketoprofen, acemetacin and etoricoxib) were more frequently prescribed before and after hospitalization. The majority of these are probably drug treatments for patients with musculoskeletal and connective tissue disorders, which make up a large part of the study population. Pain management is the central part of the treatment both before and after musculoskeletal interventions [39–41]. That these medications may be prescribed inappropriately is well documented in the literature [42–45] and their prescription is recommended only after a sound consideration of the benefits and risks. Nonetheless, this finding is intended to raise awareness particularly among healthcare providers outside the hospital setting regarding the prescribing practices of anti-inflammatory and anti-rheumatic PIMs. Furthermore, medications used to treat sleep disorders and constipation were discontinued (paraffin, zolpidem, and sodium picosulfate) after hospitalization. This might indicate treatment success.

Contrary to PIMs, the prevalence of PIMCs already decreased after hospital admission. However, previous German studies observed opposing effects of a hospitalization on PIMC prescriptions; Köhler et al. found that patients who had at least one DDI prescription amounted to 55.6% before hospital admission and 60.4% after hospital discharge [46]. Moreover, Vonbach et al. showed that patients received on average 0.59 drug-drug interactions at hospital admission, and 0.6 at hospital discharge [47]. There are several possible reasons for these different findings. First, both German studies are based on significantly smaller sample sizes including 169 and 851 patients, respectively. Second, both studies used Pharmavista software for drug-drug interaction identification. Third, Köhler et al. used a very specific patient population that is at higher risk of PIMs/PIMCs even after hospitalization due to their complex medication needs, which is consistent with the results of the ZIP regression model.

Taken together, our results indicate an increased PIM, but a decreased PIMC prevalence during hospitalization as compared to the pre- and post-hospitalization period. The variation in PIMs might be attributed to the deficient communication between hospitals and general practitioners [48, 49], different prescribing attitudes, habits, preferences and affinity toward evidence-based prescribing [50], different management and different awareness of risks related to multimorbidity and polypharmacy [51, 52], or different availability and pricing of medications in the hospital setting, for example, discounting of certain drugs that would be more expensive when prescribed in the outpatient setting [53]. Otherwise, our finding of decreasing PIMC prevalence during hospitalization could be attributed to the fact that hospital admission may give rise to reconsider and readjust all diagnoses and therapies. Presumably, further factors such as interprofessional collaboration, technical support, integration of pharmaceutical expertise might contribute to prevent PIMCs in the hospital setting. Consultations with general practitioners after hospital discharge might further improve medication safety compared to the pre-hospitalization period as changes in medications due to hospitalization are evaluated and further patients’ needs are assessed and, therefore, medication profiles might be adjusted.

As far as determinants of PIM and PIMC prescriptions are concerned, we assessed risk factors of inappropriate medications which were associated with increased numbers of PIMs and PIMCs such as older age for both, men and women. Overall these findings are in accordance with findings reported by most of the previous studies [5, 54–57]. The increase in PIMs and PIMCs might result from higher medical needs as aging evokes physiological changes and increasing likelihood of comorbid conditions. Moreover, these findings suggest that recommendations and guidelines to discontinue prescriptions of certain medications in the elderly population often do not discourage PIM and PIMC prescriptions. Our estimates of sex-related differences concerning the occurrence and frequency of prescribing PIMs are consistent with a large body of literature [6, 58, 59]. Another strand of the literature focuses on differences between men and women with regard to PIMC prescriptions. A Swiss study found that higher potential drug-drug interaction frequency was associated with female gender [22]. Similarly, a Brazilian study showed that 32.6% of men, but a substantially higher female proportion (49.2%) received such prescriptions [60]. Our varying findings could be attributed to the fact that men prevailed in diagnosis groups presumably receiving PIMCs such as diseases of the circulatory system.

Our analysis showed that people diagnosed with neoplasia, diseases of the digestive, urogenital, and the musculoskeletal system and connective tissue had higher chances of receiving at least one PIM. These morbidities were shown to be positively related to PIMs because their corresponding treatment was associated with a higher risk of PIM use [11]. For instance, medications treating nausea are predominantly prescribed in patients diagnosed with illness of the digestive system. Furthermore, patients with some injuries and poisoning might receive drugs for pain control, such as diclofenac. Patients diagnosed with diseases of the circulatory system were associated with increased likelihood of receiving at least one PIMC and the number of PIMCs (such as acetylsalicylic acid and phenprocoumon). The high prevalence of PIMs used for the treatment of patients with diseases of the circulatory system stresses the need for interventions as these diseases are the leading cause of death in Switzerland, and medical need to treat these diseases is correspondingly high [61].

We observed regional differences in probabilities of receiving at least one PIM and the number of PIMC prescriptions. Coherently, the Helsana drug report 2019 found regional differences in costs and the amount of prescribed medications. Those cantonal discrepancy might be driven by the different patient characteristics and higher density of medical services in urban regions as well as due to cultural differences [13, 62, 63]. This might suggest that regional and cultural differences result in different dispensing practices which also includes dispensing of single inappropriate and possibly interacting medications. Nevertheless, these differences in language regions in the occurrence of PIMs and number of PIMCs should be interpreted cautiously since observations of French speaking patients were scarce and, therefore, results may not be generalizable to the total French speaking hospitalized population.

Regarding the type of health insurance plan, prescriptions of PIMs and PIMCs were less frequent in patients enrolled in a managed care model. Our findings are in line with a previous Swiss study which identified a decreased PIM prevalence in patients aged 65 years and older in managed care plans (18.6%) compared to the general fee-for-service delivery models (21.1%) between 2008 and 2012 [64]. Correspondingly, a Taiwanese study showed that integrated care such as managed care plans reduced the risk of PIMC prescriptions [65]. These integrated care plans aim to ensure structured and mandatory collaboration between different healthcare providers and professions across the entire care pathway.

PIM and PIMC prescriptions were increased in patients with a low deductible. However, these findings have to be interpreted with caution since deductible choice might be based on different motivations: high deductibles might be preferred by wealthy individuals as they have the sum at their disposal as well as by less wealthy individuals in order to obtain low premium [66]. Moreover, deductible choice might be driven by risk-taking behavior [67] or low healthcare cost prediction in the following year, e.g. in individuals with less chronic diseases [68]. Another factor mitigating the practical relevance of this difference is the low statistical power in the high deductible group (*n* = 622, 11%). Thus, slight variations between the characteristics of high deductible group (e.g., morbidity) may result in different significance levels. The deductible variable is important nationally and is used to control for differences between the insurance collectives. However, its results should not be overinterpreted [69].

Several limitations of this study deserve consideration. First, the study population consisted of people who were hospitalized in a large private hospital group and who were insured by one leading health insurance company. Therefore, prescription patterns of inappropriate medications might differ in other settings and hospital groups which reduces the generalizability of the results. To address this, we controlled for a comprehensive set of variables in the calculated models, such as sociodemographic variables (age, gender), insurance variables (managed care, height of deductible, and supplementary hospital insurance), and medical diagnosis. Furthermore, Helsana insurance can be chosen by all patient groups irrespective of health status and region. Second, our study observed overall changes in PIM and PIMC prescriptions regardless of patient diagnosis. Thus, the documented changes in inappropriate medication prescriptions after hospitalization are generally to be expected, although this may hold for individual medical conditions. This leaves the opportunity for future studies to further analyze changes in medication prescriptions for specific medical conditions across the continuum of care. Nonetheless, the present study provides insight into the overall changes in potentially inappropriate medications after hospitalization. In our analysis, we used the PRISCUS list. This list does not target specific patients such as frail persons. Similarly, the list used to derive PIMCs does not differentiate between different target populations. Moreover, in the context of the present study we did not consider different degrees of the severity of the inappropriate medications and inappropriate medication interaction. Furthermore, these lists are general recommendations not meant to overrule clinical assessment in specific clinical situations [70]. However, our study gives insight into the prescription patterns of medications that are likely to be harmful because of the high risk of adverse drug events, according to expert consensus. In addition, only inpatient diagnoses were used in this study, although medial conditions may change over time. Thus, changes in diagnoses were not considered and might have affected medication prescriptions. Future studies should take into account further factors that presumably influence PIM and PIMC prescriptions such as hospitalization duration, number of service providers involved, dispensing channels, or incentives for PIM and PIMC prescriptions. Empirical research highlights medication safety as the major cause of concern of the Swiss healthcare system as medication errors are common. The federalist system delegates the healthcare planning to the autonomy of the cantons and curbs national healthcare reforms. Thus, no national strategy explicitly targets medication safety. Nevertheless, an increasing number of national activities are dedicated to various aspects of medication safety such as the “progress!” programme within the National Quality Strategy [71]. The Swiss National Science Foundation is involved in medication safety in some research projects in the setting of the National Research Programme “Smarter Health Care” (NRP 74) [72]. Another strategy that has been acknowledged by several international patient safety organizations such as the Institute for Healthcare Improvement (IHI) [73], The Joint Commission (TJC) [74] and the World Health Organization (WHO) [75] to reduce medication errors that may lead to adverse drug events is medication reconciliation. Fundamentally, medication reconciliation consists of identifying unintended medication discrepancies, especially at transitions in healthcare [76, 77]. However, there is variability in medication reconciliation practices, due to heterogeneous guidance on how to perform the process. One possible implementation tool for medication reconciliation could be the electronic patient file, which is currently being debated in Switzerland. This record increases transparency of medical care provision independently of the healthcare setting as it allows physicians to digitally track medication prescriptions and to have the possibility to adapt their prescribed medications to the patients’ needs [78, 79]. Many other recent approaches tried to increase drug safety such as the development of lists and tools for the identification and reduction of problematic drugs [80, 81], educational programs [82], comprehensive geriatric assessment or geriatric care teams [83], information and communication technology interventions [84, 85] or medication review by different types of health professionals [86]. Thus, according to the most recent research the inclusion of clinical pharmacists in interventions might yield benefits in medication safety after hospital discharge (i.e. medication reconciliation, in-home assessment by a clinical pharmacist, evidence-based educational resources, communication with the primary care team, and telephone follow-up) [77, 87]. Another promising approach is blockchain technology. It is widely used in the financial industry and not established yet in the healthcare sector. Due to the sound theoretical foundation, blockchain technology does allow for improved data security and increased transparency [87, 88]. Thus, it might serve as an instrument which enables networking between doctors, health insurance companies and patients, thus enabling therapies and medications to be coordinated more efficiently and duplicate examinations to be avoided [89]. However, these interventions and technologies have not succeeded yet in reducing problematic medications in the community-dwelling population in Switzerland, partly because great skepticism prevails with regard to sensitive data. Therefore, a comprehensive national strategy involving all stakeholders is exigent for the Swiss health system to ensure medication safety across all healthcare settings.

## Conclusions

Overall, PIM and PIMC prevalence was relatively high in this study population. Hospitalizations were shown to offer a chance to adjust medication safety as PIMC prescriptions were reduced after hospital discharge. The present study also highlights important determinants of medication safety, such as age, being in a managed care model, and diagnosis. In addition, the present study quantifies the burden of inappropriate medications and, thus, stresses the need for interventions to prevent avoidable adverse drug reactions. Furthermore, this study supports the allocation of resources in planning instruments enhancing medication safety by providing information on factors influencing drug safety in the Swiss healthcare system.

## Data Availability

The data that support the findings of this study are available from Helsana (https://www.helsana.ch/en/helsana-group). Restrictions apply to the availability of these data, which were used under license for the current study and therefore are not publicly available. However, data are available from the authors upon reasonable request and with permission of Helsana (gesundheitskompetenz@helsana.ch).

## Abbreviations

PIM: Potentially inappropriate medication
PIMC: Potentially inappropriate medication combination
ATC: Anatomical Therapeutic Chemical
DDI: Drug-drug interactions
